# Interleukin-8/CXCR1 Signaling Contributes to the Progression of Pulmonary Adenocarcinoma Resulting in Malignant Pleural Effusion

**DOI:** 10.3390/cells13110968

**Published:** 2024-06-03

**Authors:** Yi-Ming Chang, Wen-Yen Huang, Shih-Hsien Yang, Chia-Ing Jan, Shin Nieh, Yaoh-Shiang Lin, Su-Feng Chen, Yu-Chun Lin

**Affiliations:** 1 Graduate Institute of Medical Sciences, National Defense Medical Center, Taipei 11490, Taiwan; ymjhang@vghks.gov.tw (Y.-M.C.); thero0823@mail.ndmctsgh.edu.tw (S.-H.Y.); 2 Department of Pathology and Laboratory Medicine, Kaohsiung Veterans General Hospital, Kaohsiung 813414, Taiwan; ci@vghks.gov.tw; 3 Department of Radiation Oncology, Tri-Service General Hospital, National Defense Medical Center, Taipei 11490, Taiwan; hwyyi@ndmctsgh.edu.tw; 4 Office of General Affairs and Occupational Safety, National Defense Medical Center, Taipei 11490, Taiwan; 5 Department of Pathology, Tri-Service General Hospital, National Defense Medical Center, Taipei 11490, Taiwan; ns1014@mail.ndmctsgh.edu.tw; 6 Department of Otorhinolaryngology, Head and Neck Surgery, Kaohsiung Veterans General Hospital, Kaohsiung 813414, Taiwan; yaohshiang@vghks.gov.tw; 7 Department of Dentistry, School of Dentistry, China Medical University, Taichung 404333, Taiwan

**Keywords:** interleukin-8, CXCR1, interleukin-8/CXCR1 signaling, fluid microenvironment, epithelial-mesenchymal transition, cancer stem cell

## Abstract

Pulmonary adenocarcinoma (PADC) treatment limited efficacy in preventing tumor progression, often resulting in malignant pleural effusion (MPE). MPE is filled with various mediators, especially interleukin-8 (IL-8). However, the role of IL-8 and its signaling mechanism within the fluid microenvironment (FME) implicated in tumor progression warrants further investigation. Primary cultured cells from samples of patients with MPE from PADC, along with a commonly utilized lung cancer cell line, were employed to examine the role of IL-8 and its receptor, CXCR1, through comparative analysis. Our study primarily assessed migration and invasion capabilities, epithelial-mesenchymal transition (EMT), and cancer stem cell (CSC) properties. Additionally, IL-8 levels in MPE fluid versus serum, along with immunohistochemical expression of IL-8/CXCR1 signaling in tumor tissue and cell blocks were analyzed. IL-8/CXCR1 overexpression enhanced EMT and CSC properties. Furthermore, the immunocytochemical examination of 17 cell blocks from patients with PADC and MPE corroborated the significant correlation between upregulated IL-8 and CXCR1 expression and the co-expression of IL-8 and CXCR1 in MPE with distant metastasis. In summary, the IL-8/ CXCR1 axis in FME is pivotal to tumor promotion via paracrine and autocrine signaling. Our study provides a therapeutic avenue for improving the prognosis of PADC patients with MPE.

## 1. Introduction

Pulmonary adenocarcinoma (PADC), the most common type of lung cancer, presents significant challenges due to its aggressive nature and propensity to metastasize. One of the severe complications associated with advanced stages of this malignancy is malignant pleural effusion (MPE), a condition characterized by the accumulation of fluid in the pleural cavity, leading to respiratory distress and diminished quality of life in affected patients [1]. Understanding the molecular mechanisms driving the progression of PADC and the development of MPE is crucial for improving therapeutic strategies and patient outcomes.

Interleukin-8 (IL-8), a pro-inflammatory cytokine, has been implicated in various aspects of cancer progression, including angiogenesis, tumor cell proliferation, and metastasis [2,3,4]. IL-8 exerts its biological effects primarily through binding to its cognate receptors, CXCR1 and CXCR2 [5], which are expressed on the surface of various cell types [6,7,8,9,10], including cancer cells and cells within the tumor microenvironment [3]. The IL-8/CXCR1 signaling axis, in particular, has garnered attention for its role in enhancing the malignant phenotype of cancer cells [11]. Recent studies have demonstrated that IL-8/CXCR1 signaling contributes to the progression of PADC by promoting tumor cell survival, invasion, and resistance to apoptosis [11,12,13]. Moreover, this signaling pathway has been associated with the induction of epithelial-mesenchymal transition (EMT), a process whereby epithelial cells acquire mesenchymal characteristics, enhancing their migratory and invasive capabilities. EMT is a critical step in the metastatic cascade, facilitating the dissemination of cancer cells from the primary tumor site to distant organs and the pleural cavity, where they contribute to the formation of MPE [1]. In addition to EMT, anoikis resistance is another crucial factor in the metastatic process. Anoikis, a form of programmed cell death that occurs when cells detach from the extracellular matrix, acts as a barrier to metastasis [14]. However, cancer cells that develop resistance to anoikis can survive during detachment, circulate through the body, and colonize distant sites. This resistance is pivotal in the metastatic potential of PADC, allowing tumor cells to thrive in the pleural cavity and contribute to MPE formation.

The IL-8/CXCR1 axis not only supports tumor growth and metastasis but also creates a pro-tumorigenic microenvironment by modulating the behavior of immune cells within the tumor milieu [3,15]. The resultant chronic inflammatory state further exacerbates tumor progression and the development of MPE. Adding to the complexity of metastasis is the “seed and soil” theory [16,17], which posits that metastatic spread depends on the interaction between disseminating tumor cells (the “seed”) and the receptive microenvironment (the “soil”) of distant organs. The IL-8/CXCR1 signaling pathway exemplifies this theory, as it not only enhances the intrinsic aggressiveness of tumor cells but also conditions the microenvironment to support their growth and survival.

The mechanism that allows metastatic PADC cells in MPE to survive and proliferate involves the pleural fluid, which is rich in growth factors and cytokines [18], particularly IL-8. Depletion of IL-8 reduces both stemness and tumorigenicity of colorectal cancer cells, underscoring its pivotal role in maintaining malignancy [19]. IL-8 also regulates the migration and growth of glioblastoma CSCs, and inhibition of CSC CXCR2 signaling abolishes their stimulatory effects on brain endothelial cells, hindering tumorigenesis in vivo [20]. Drug-induced IL-8 signaling confers chemotherapeutic resistance [4], suggesting that inhibiting IL-8 may be a significant therapeutic intervention for the fluid microenvironment (FME).

The aim of this study is to highlight the significance of IL-8/CXCR1 signaling in the pathogenesis of PADC and its role in the occurrence of MPE. Elucidating these molecular mechanisms may mitigate tumor progression and improve the prognosis for patients suffering from this devastating condition. Our research model of pleural fluid allows investigation of IL-8 in tumor progression and cancer stemness, potentially leading to IL-8 targeting as a therapeutic strategy for MPE patients.

## 2. Materials and Methods

### 2.1. Collection of Clinical Effusion, Serum, and Tissue Specimens

Clinical effusion and serum from a total of 77 cases were collected, of which 19 were excluded due to incomplete data or insufficient specimens. Further exclusions were made for cases where the time difference between serum collection and pleural effusion exceeded 24 h, resulting in a reduction in serum group sizes. Consequently, the study group, initially comprising 33 patients diagnosed with PADC with MPE, was reduced to 16 for the serum analysis. Meanwhile, the control group, which initially included 25 patients with systemic diseases with pleural effusion (SDPE) such as congestive heart failure, cirrhosis of the liver, or other systemic conditions, was reduced to 23 for serum analysis. Clinical data are summarized in Table S1. These cases were retrieved from the archives of the Department of Pathology at Tri-Service General Hospital in Taipei, Taiwan, covering the period from 2015 to 2020, with permission from the institutional review board (protocol code: C202005184). The MPE group underwent histopathological and cytological analysis using TTF-1 immunohistochemical staining.

Tissue specimens from a total of 56 patients with PADC were initially collected from the archives of the Kaohsiung Veterans General Hospital, Kaohsiung, Taiwan with the permission of the institutional review board (protocol code KSVGH22-CT9-08), spanning 2013 to 2018, all of whom had surgical specimens and cell blocks, which are rare because these cases involved PADC with MPE, typically indicative of advanced-stage cancer. Often, such patients do not have surgical specimens available as diagnoses are usually made through MPE or small biopsies followed by a series of molecular tests (e.g., EGFR, PDL1, ROS1, ALK, and so on). Although surgical specimens were available for these 56 cases, 17 were excluded due to loss of follow-up or lack of complete clinicopathological data. Among the remaining cases, only 17 had adequate tumor cell numbers in their cytospins and cell blocks. Follow-up for all study cases was conducted over five years. Clinical data are summarized in Table S2.

### 2.2. Origins of the Two Selected Cell Lines

NCI-H1792 (H1792), derived from a human lung adenocarcinoma, was obtained from the American Type Culture Collection (ATCC). Exhibiting epithelial morphology, the cell line was isolated from a 50-year-old white male patient with PADC and has been widely utilized in cancer research. For more information, please visit https://www.atcc.org/products/crl-5895, accessed on 16 March 2024. Another primary cultured cell line (P#2045) was prospectively collected using a nonadhesive culture system [21] from a 62-year-old male patient diagnosed with PADC with MPE, identified as the most suitable cell line among eight established primary culture cells, and further confirmed histologically and cytologically using TTF-1 (Figure S1). Conventional and primary cultures of MPE were conducted as described in our previous study [1,21].

### 2.3. Immunohistochemistry, Immunocytochemistry, and Immunofluorescence

Thirty-three patients with PADC and MPE recruited from Kaohsiung Veterans General Hospital between 2015 and 2020 were included in this study. Among these cases, 16 were excluded due to a lack of surgically removed specimens, and 17 cases with adequate clinical and pathological information for IHC studies of IL-8 and CXCR1, were included to the study. Staining intensity was classified as absent (0), mild (1+), moderate (2+), or strong (3+) [22]. Intensities greater than 1, including 1, 2, and 3, were defined as positive, while zero was regarded as negative. NCI-H1792 and P#2045 were used for IF studies, with TTF-1 confirming the presence of PADC in surgical specimens and cell blocks. All antibodies used in this study are listed in Table S3. These methods were performed as described in our previous study [21,23,24].

### 2.4. Cell Viability Assay

Cells were seeded in a 96-well plate at a density of 5000 cells/well for the MTT assay. Treatments with different concentrations of IL-8 (0, 5, 20, and 50 μg/mL) (Sigma-Aldrich, St. Louis, MO, USA) were administered 12 h after seeding. Cell viability was determined on days 1, 2, and 3 after treatment by adding 20 μL of MTT solution (5 mg/mL in PBS) (Sigma-Aldrich). Cells were incubated in MTT solution for 4 h at 37 °C. Subsequently, the culture medium was removed and replaced with 150 μL of DMSO to dissolve the crystals. Samples were incubated at room temperature for 10 min, and absorbance was measured at 568 nm using a microplate reader (Bio-Rad Laboratories, Hercules, CA, USA), as described previously [21,23,24]. Experiments were conducted in triplicate.

### 2.5. Western Blot Analysis

Cell lysates were prepared in RIPA lysis buffer containing 50 mM Tris-HCl (pH 7.4), 150 mM NaCl, 1% Triton X-100, 1% sodium deoxycholate, 0.1% SDS, 1 mM PMSF, and a protease inhibitor cocktail. Protein samples were loaded and separated on a 10% sodium dodecyl sulfate-polyacrylamide gel and transferred to a polyvinylidene difluoride (PVDF) membrane (Millipore, Billerica, MA, USA). The membranes were washed with Tris-buffered saline (TBS), blocked with 5% (*w*/*v*) nonfat milk in TBS-Tween20 (1%, *v*/*v*, TBST) for 1 h at room temperature. After three washes of 5 min each with TBST, membranes were incubated with primary antibodies (1:1000) in TBST with 5% (*w*/*v*) bovine serum albumin (BSA) overnight at 4 °C. After three washes with TBST, the membranes were incubated with horseradish peroxidase-conjugated secondary antibodies (1:5000) for 1 h at room temperature. We employ tubulin as a loading control in Western blot (WB) analysis to demonstrate the consistency of protein content across samples. This practice ensures the comparability and accuracy of our results. For each batch of test samples, we first verify the expression of tubulin to maintain uniform loading amounts and confirm successful protein transfer to the transfer membrane. We adhere to the standard Western blot protocol. In each of our experiments, we run two gels: one for tubulin, serving as the standard, and another for the target protein. Tubulin typically requires only about 10 s of exposure time due to its rapid color development, while the other target protein requires varying exposure times. The bands were detected using an enhanced chemiluminescence (ECL) detection kit (Thermo Electron Corp., Rockford, IL, USA). The relative quantity of proteins was analyzed by Image Gauge^®^ Ver. 4.0 software (Fuji Film, Tokyo, Japan) and normalized to the loading controls, as described previously [21,23,24]. All antibodies used in this study are listed in Table S3. Experiments were conducted in triplicate.

### 2.6. In Vitro Migration and Invasion Assay

Cell migration and invasion used lung cancer cell lines NCI-H1792 and MPE cell line P#2045, cultured in RPMI-1640 medium supplemented with 10% fetal bovine serum (FBS) and maintained at 37 °C in a 5% CO_2_ environment until reaching logarithmic growth. Migration and invasion assays were conducted using Transwell inserts (Corning Costar, Corning, NY, USA) with polycarbonate membranes (8-μm pore size) in 24-well plates. For the invasion assays, filters were coated with 20 µg/mL Matrigel (Becton-Dickinson, Franklin Lakes, NJ, USA) overnight at 4 °C to simulate extracellular matrix barriers. A total of 100 µL of cell suspension at 1.0 × 10^5^ cells/mL in a serum-free medium containing specified compounds was seeded into the upper chamber, while the lower chamber received 800 µL of the culture medium with 10% FBS and similar treatment compounds. The setups for migration assays mirrored those for invasion but without the Matrigel coating. After 24 h of incubation, non-migratory or non-invading cells were removed from the upper membrane surface with a cotton swab. Migrated or invaded cells on the lower membrane surface were fixed with methanol, stained with crystal violet, rinsed, and air-dried. The evaluation involved photographing the stained cells and counting them under an inverted microscope across ten random fields at 200× magnification. Both assays were repeated three times, counting cells in five random fields per experiment. Results were expressed as a fold increase over the control group, and statistical analysis was performed using a Student’s *t*-test, considering a *p*-value of <0.05 as statistically significant [21,23,24].

### 2.7. In Vitro Wound-Healing Assay

The wound-healing assay starts with the culture of lung cancer cell lines NCI-H1792 and MPE cell line P#2045. These cells were cultured in RPMI-1640 medium enriched with 10% fetal bovine serum (FBS) and maintained in a controlled environment at 37 °C with 5% CO_2_ until near-confluence was achieved, setting the stage for the scratch assay. The creation of the scratch was performed using a sterile 200 μL pipette tip to draw a straight line across the cell monolayer, mimicking a wound. Following the scratch, cells were washed multiple times with 1× PBS to eliminate loose and dead cells, ensuring a clean area for observing migration. To focus on migration rather than proliferation, cells were then incubated in RPMI-1640 supplemented with a reduced FBS concentration of 1%, and images of the scratch were captured at 0 h, and subsequently at 12 or 24 h using the same field of view to maintain consistency. Image analysis was conducted using an inverted microscope, and the scratch width was quantitatively assessed using ImageJ software (Version 1.53K). The percentage of wound closure was calculated to evaluate the migratory capacity of the cells, with the formula: [(initial width *−* final width)/initial width] × 100%. The experiments were repeated in three technical replicates. Results were presented as the percentage of scratch closure, and statistical significance between the control and treatment groups was determined using a Student’s *t*-test.

### 2.8. Enzyme-Linked Immunosorbent Assay

The level of soluble IL-8 secreted into the cell culture medium was determined using an enzyme-linked immunosorbent assay (ELISA; Sigma-Aldrich, St. Louis, MO, USA) according to the manufacturer’s instructions. In brief, related reagents and samples were prepared, and the samples containing IL-8 were added to each well at 100 μL. After 90 min of incubation, the plates were incubated with biotinylated antibodies. Immunoreactivity was determined using an avidin-horseradish peroxidase (HRP)-TMB assay. The reactions were stopped by the addition of a TMB stop buffer, and absorbance was measured at 450 nm using a microplate reader (Bio-Rad 680). A curve of absorbance versus the concentration of the standard wells was plotted. Experiments were conducted in triplicate. The concentration of human IL-8 in the tested samples was determined by comparing the absorbance of the samples to a standard curve, as described previously [21,23,24].

### 2.9. Sphere Culture

Cells were cultured in plastic culture ware with a nonadhesive surface. A 10-cm dish was coated with agarose thin films to create a nonadhesive environment. Cells were seeded at a density of 5 × 10^4^ live cells/10 cm dish, and the culture medium was changed every alternate day until sphere formation, as described previously [21,23,24].

### 2.10. Assays for Chemosensitivity and Radiosensitivity

Cells were seeded in a 10 cm dish at a density of 1 × 10^6^ cells/dish. For the chemosensitivity assay, cells were exposed to varying concentrations (10, 20, 30, 40, and 50 µM) of Cisplatin (Sigma, St. Louis, MO, USA) for 48 h. For the radioresistance assay, cells were irradiated using Cs-137 to deliver different doses (2, 4, 6, 8, and 10 Gy). The relative surviving fraction of cells was determined using the MTS assay with the CellTiter 96 Aqueous One Solution Cell Proliferation Assay kit (Promega, Madison, WI, USA) after 36 h of radiation treatment, as described previously [21,23,24]. Experiments were conducted in triplicate.

### 2.11. Statistical Analysis

The independent Student’s *t*-test, paired *t*-test, or ANOVA was employed to compare continuous variables between groups, while the Χ^2^ test was utilized for dichotomous variables. The level of statistical significance was set at *p* < 0.05. All statistical analyses were performed using SPSS version 20 software (SPSS Inc., Chicago, IL, USA).

## 3. Results

### 3.1. IL-8 Levels in MPE and Serum of PADC Was Higher Than Those in Systemic Disease with Pleural Effusion (SDPE) and IL-8 Levels in Both Representative Cell Lines Were Compared

**Figure 1:**
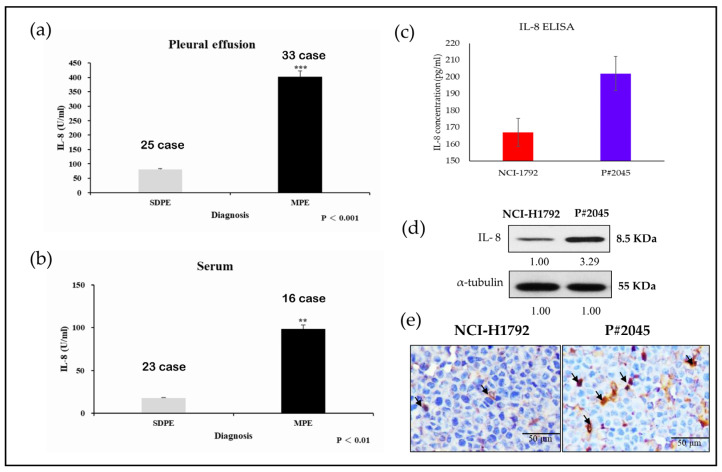
Comparative analysis of IL-8 levels in pleural effusion and serum, with further comparisons of IL-8 in two representative cell lines. (**a**) High levels of IL-8 were observed in PADC with MPE which were statistically higher than those in SDPE (*** *p* < 0.001, Student’s *t*-test) (Experiments were conducted in triplicate). (**b**) Similar results were found with higher IL-8 levels in the serum of patients with PADC and MPE, compared to that in patients without MPE (** *p* < 0.01, Student’s *t*-test). (Experiments were conducted in triplicate). Interestingly, higher IL-8 levels were found in pleural effusion compared to serum. (**c**) Comparison of IL-8 levels in the culture media of P#2045 and NCI-1792 showed different levels, with a higher level of IL-8 noted in MPE (P#2045) by ELISA (Experiments were conducted in triplicate). (**d**) IL-8 protein was observed in both cell lines, with higher levels noted in MPE (P#2045) by Western blotting (Experiments were conducted in triplicate). (**e**) Stronger expression of IL-8 was found in MPE (P#2045) compared to PADC (NCI-1792) by ICC (arrows indicate positive IL-8 findings) (magnification, ×400, Scale bar: 50 μm).

33 patients with PADC and corresponding MPE were included in our study, and 25 patients of systemic disease with pleural effusion (SDPE) without evidence of PADC were included in the control group. Higher IL-8 levels were observed in PADC patients with MPE compared to those treated with SDPE (*p* < 0.001) (Figure 1a). Additionally, elevated serum IL-8 levels were detected in 16 patients with PADC and MPE compared to 23 patients with SDPE (*p* < 0.01) (Figure 1b). Furthermore, we conducted a paired t-test on effusion samples (n = 25) and serum samples (n = 10). The statistical outcomes show similar results and are shown in Figure S2 and Table S4. Importantly, IL-8 levels were over fourfold higher in the pleural fluid than in the serum, consistent with previous studies [6]. Comparative analysis of the chosen cell lines revealed significantly higher IL-8 levels in P#2045 [1] a primary cultured cell line with continuous exposure to the fluid microenvironment (FME), compared to NCI-H1792 from PADC without MPE. Furthermore, IL-8 levels in both cell lines were detected in culture media using ELISA and Western blotting (Figure 1c,d), with differences confirmed by ICCs (Figure 1e). These results suggest a pivotal role for IL-8 in MPE associated with FME.

### 3.2. IL-8 Promoted Epithelial-Mesenchymal Transition (EMT) and Activities of Migration and Invasion While Increasing the Expression of EMT-Associated Markers

**Figure 2:**
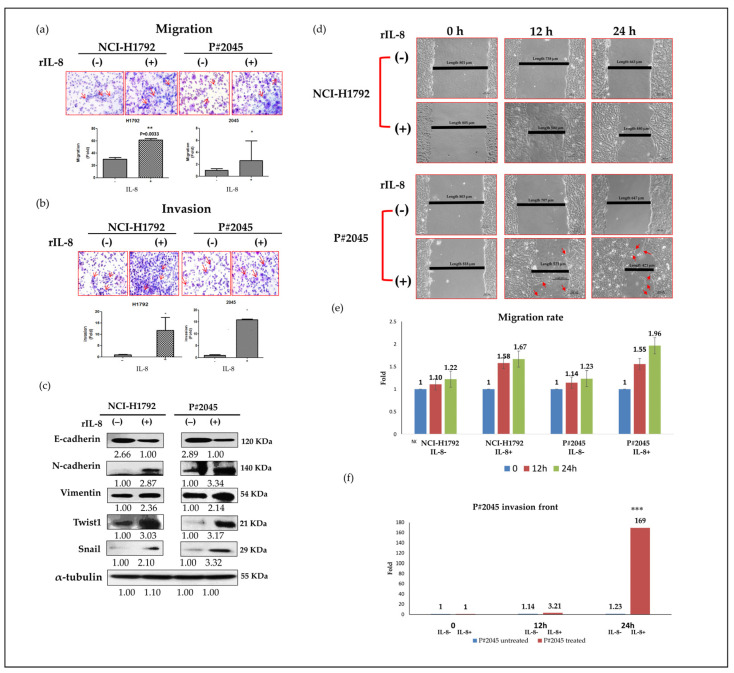
Paracrine effect of IL-8 via the administration of rIL-8 enhanced migration and invasion activities, and increased EMT properties. (**a**) Migration assays showed that rIL-8 significantly enhanced migration properties in NCI-H1792 and P#2045 cell lines (** *p* < 0.01 and * *p* < 0.05, respectively, Student’s *t*-test) (Experiments were conducted in triplicate) (The red arrows indicate tumor cells). (**b**) Invasion assays revealed that rIL-8 significantly enhanced invasion properties in NCI-H1792 and P#2045 cell lines (* *p* < 0.05 and * *p* < 0.05, respectively, Student’s *t*-test) (Experiments were conducted in triplicate) (The red arrows indicate tumor cells). (**c**) Treatment with rIL-8 resulted in enhanced EMT properties in both cell lines, including decreased expression of E-cadherin, increased expression of N-cadherin, vimentin, and transcription factors Twist-1 and Snail (Experiments were conducted in triplicate). (**d**) The wound-healing assay experiment at different time points, 0, 12, and 24 h in both NCI-H1792 and P#2045 cell lines. The results indicate that compared to the cell line NCI-H1792, the distance between P#2045 cells decreased by 50% within 12 h, and by 24 h, the cells on both sides had already connected. This wound-healing assay resulted in the significant finding that P#2045 cells were easily migrated to the scratched center, indicating an increase of migratory activities compared to NCI-H1792 cells (magnification, ×400, Scale bar = 100 μm) (Experiments were conducted in triplicate). (**e**) In the wound-healing assay, after exposure to rIL8 for 12 and 24 h, cell migration in the NCI-H1792 cell line increased by 1.58-fold and 1.66-fold, respectively. In the P#2045 cell line, the migration increased by 1.55-fold at 12 h and 1.96-fold at 24 h. (**f**) In the wound-healing assay, the P#2045 cell line treated with rIL8, the increase in the invasive front was observed to be 3.2-fold at 12 h and 169.2-fold at 24 h (*** *p* < 0.001, Student’s *t*-test).

Given the higher levels of IL-8 observed in patients with MPE from PADC (P#2045), our objective was to utilize recombinant IL-8 (rIL-8) to treat two representative cell lines, NCI-H1792 and P#2045, mimicking paracrine IL-8 signaling cascades, using a previously established in vitro model of pleural fluid [21]. The optimal reaction time was 48 h with a plateau concentration of 20 ng/mL (Figure S3). The migratory assay and invasive assay, performed in triplicate, show IL-8 significantly enhanced cell migration (NCI-H1792, *p* < 0.01 and P#2045, *p* < 0.05, Figure 2a) and invasion (NCI-H1792, *p* < 0.05 and P#2045, *p* < 0.05, Figure 2b). Comparative analysis revealed alterations in EMT-associated markers induced by IL-8, including downregulation of E-cadherin and upregulation of N-cadherin, vimentin, Twist-1, and Snail (Figure 2c). The wound-healing assay, performed in triplicate, aimed to evaluate cellular migration in the NCI-H1792 and P#2045 cell lines at 0, 12, and 24 h post-scratch (Figure 2d). Results consistently demonstrated that rIL8 significantly enhances cell migration. Initially, no significant migration was observed in the NCI-H1792 cell. However, after 12 h of rIL8 treatment, there was a noticeable 1.58-fold increase in migration, escalating to a 1.66-fold increase at 24 h relative to untreated controls (Figure 2d, Table S5). Similarly, the P#2045 cell line exhibited no significant baseline migration but showed a marked 1.57-fold increase at 12 h post-rIL8 treatment, which further intensified to a 1.96-fold increase at 24 h (Figure 2d, Table S5). Moreover, the migratory P#2045 cells were predominantly observed at the leading edge of the invasive fronts (*p* < 0.01, Figure 2e) as highlighted by arrows in the graphical representation (Figure 2d), suggesting a more aggressive migration pattern. These findings indicate a potential difference in the intrinsic migratory capacities between the two cell lines, with P#2045 cells showing a superior ability to migrate and potentially invade compared to the NCI-H1792 cells (Figure 2d,e).

### 3.3. High Levels of IL-8 in MPE Enhanced CSC Properties, Including Sphere Formation, Increased Expression of CSC Markers, and Resistance to Radiotherapy and Chemotherapy, as Well as Drug Resistance Genes

**Figure 3:**
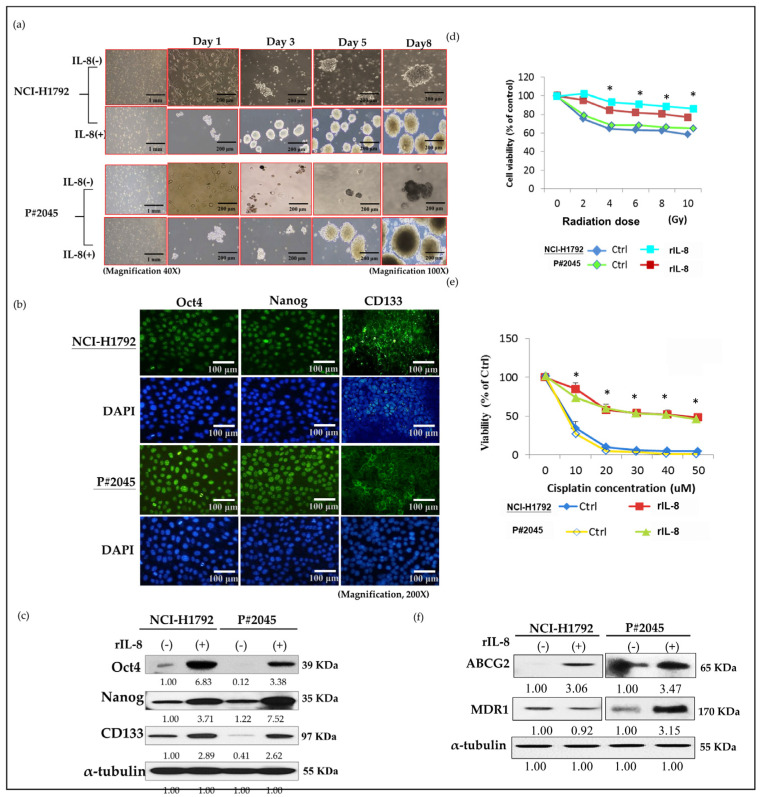
IL-8 promotion and maintenance of CSC properties: sphere formation, CSC markers, radioresistance, chemoresistance, and drug resistance. (**a**) rIL-8 enhanced sphere size and increased sphere number on different days (day 1, day 3, day 5, and day 9) in both NCI-H1792 and P#2045 (magnification, ×100). (**b**) Expression of CSC markers, including OCT4, Nanog, and CD133 in both NCI-H1792 and P#2045 was verified by immunofluorescence (magnification, ×200). (**c**) rIL-8 promoted the expression of CSC markers, including Oct4, Nanog, and CD133, in both NCI-H1792 and P#2045 by western blotting (Experiments were conducted in triplicate). (**d**) Both cell lines treated with rIL-8 showed a good survival rate in radiosensitivity assays (* *p* < 0.05, ANOVA) (Experiments were conducted in triplicate). (**e**) Both cell lines treated with rIL-8 also exhibited a good survival rate in chemosensitivity assays (* *p* < 0.05, ANOVA) (Experiments were conducted in triplicate). (**f**) Both cell lines treated with rIL-8 increased expression of ABCG2 and MDR1 (Experiments were conducted in triplicate).

In both NCI-H1792 and P#2045 cells treated with rIL-8 for 24 h, the two cell lines aggregated, merged, and initially differentiated into three-dimensional spheres, a phenomenon not observed in the control groups (Figure 3a). The spheres of both NCI-H1792 and P#2045 cells treated with r-IL-8 increased in number, compactness, and size by day 8 (Figure 3a), indicating high survival ability without tissue attachment, especially in MPE. In addition, the immunoexpression of CSC markers Oct4, Nanog, and CD133 in both cell lines was detected using immunofluorescence (Figure 3b). Treatment with rIL-8 led to a marked increase in the protein expression of CSC markers, including Nanog, CD133, and Oct4 (Figure 3c), indicating a paracrine effect of IL-8 that enhances CSC properties. Moreover, NCI-H1792 and P#2045 cells treated with rIL-8 after exposure to different doses of radiation therapy exhibited increased resistance compared to the control group (Figure 3d). Similarly, cells treated with rIL-8 following cisplatin therapy for 48 h showed enhanced chemoresistance compared to the control groups (Figure 3e). The protein expression of drug resistance genes MDR1 and ABCG2 also increased significantly in NCI-H1792 and P#2045 cell lines treated with rIL-8 (Figure 3f).

### 3.4. An Increase in CXCR1 Expression Induced by IL-8 Was Observed via Paracrine and Autocrine Signaling Pathways in MPE

**Figure 4:**
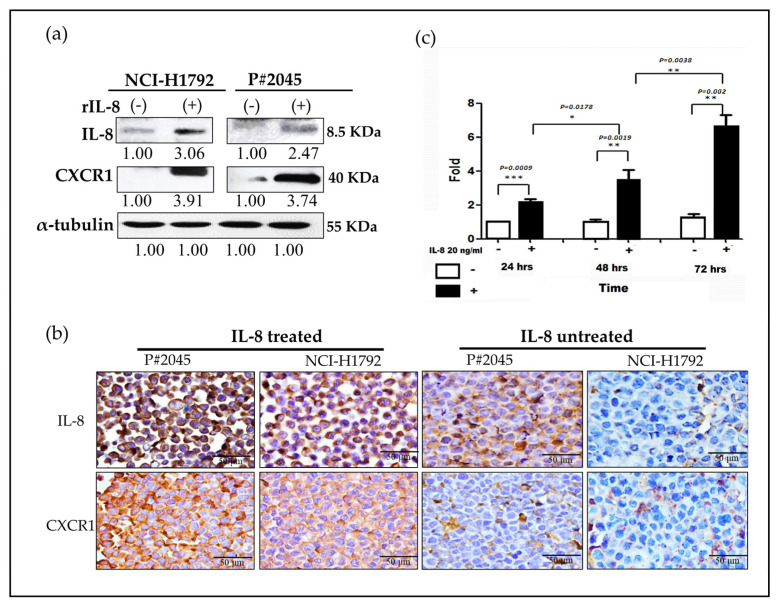
Demonstration and verification of paracrine and autocrine effects of IL-8/CXCR1. (**a**) Two representative cell lines, NCI-H1792 and P#2045, were treated with exogenous IL-8 for 24 h. Subsequent analyses were conducted using western blot and ICC to assess the effects of the treatment. Increased CXCR1 expression in both cell lines (NCI-H1792 and P#2045) following the addition of rIL-8, as shown by western blotting (Experiments were conducted in triplicate). (**b**) A marked increase in the expression of CXCR1 upon the addition of rIL-8 in both cell lines (NCI-H1792 and P#2045), was demonstrated by ICC. (magnification, ×400, Scale bar: 50 μm). Enhanced IL-8 expression was also observed in both cells using ICC (magnification, ×400, Scale bar: 50 μm). However, stronger expressions of IL-8/CXCR1 were seen in P#2045 compared to NCI-H1792 due to the long-term influence of FME, implicating the importance of FME. (**c**) A time-dependent trend showing progressively increasing levels of IL-8 was observed in P#2045 cells, implying an autocrine mechanism, which demonstrated a systematic escalation in IL-8 concentration over the course of the experiment at 24, 48, and 72 h. (Experiments were conducted in triplicate) (* *p* < 0.05, ** *p* < 0.01, *** *p* < 0.001, respectively, Student’s *t*-test).

Both NCI-H1792 and P#2045 cell lines, when treated with rIL-8, exhibited a marked increase in IL-8 and CXCR1 protein levels, indicative of stimulated paracrine signaling effects (Figure 4a). To further authenticate these observations, we utilized immunocytochemistry (ICC) on cell blocks, which corroborated the initial findings. Notably, a more pronounced increase in IL-8/CXCR1 expression was observed in P#2045 cells in comparison to NCI-H1792 cells. This significant difference strengthened the impact of the FME on cellular behavior and protein expression (Figure 4b). Additionally, a time-dependent augmentation in IL-8 levels was recorded in the P#2045 cell lines. This observation suggests the intriguing possibility of an autocrine signaling mechanism involving IL-8 and CXCR1 (Figure 4c). The gradual increase in IL-8 over time in these cell lines implies a continuous and self-sustaining signaling loop that may contribute to the aggressive nature of the tumor cells within the unique FME of MPE. As illustrated in Figure 4b, the P#2045 cell line has been conditioned by the FME and exhibits a higher expression of CXCR1, which is already influenced by the paracrine effects in MPE. The addition of rIL-8 leads to cellular proliferation within 24 h. This intervention further amplifies both autocrine and paracrine activities, activating the IL-8/CXCR1 positive feedback loop. This activation results in an increased release of IL-8 into the extracellular environment. Consequently, an elevated level of IL-8 can be detected using ELISA, implying the activation of this feedback mechanism and the resultant augmentation of IL-8 secretion, which underscores the dynamic interplay between autocrine and paracrine signaling in this context. These above patterns imply the hypothesis of both paracrine and autocrine effects.

### 3.5. IL-8/CXCR1 Immunoexpression Correlates with Clinical Outcomes in Patients with PADC and MPE Indicating MPE as an Early Metastatic Indicator for Distant Metastasis Preparation

**Figure 5:**
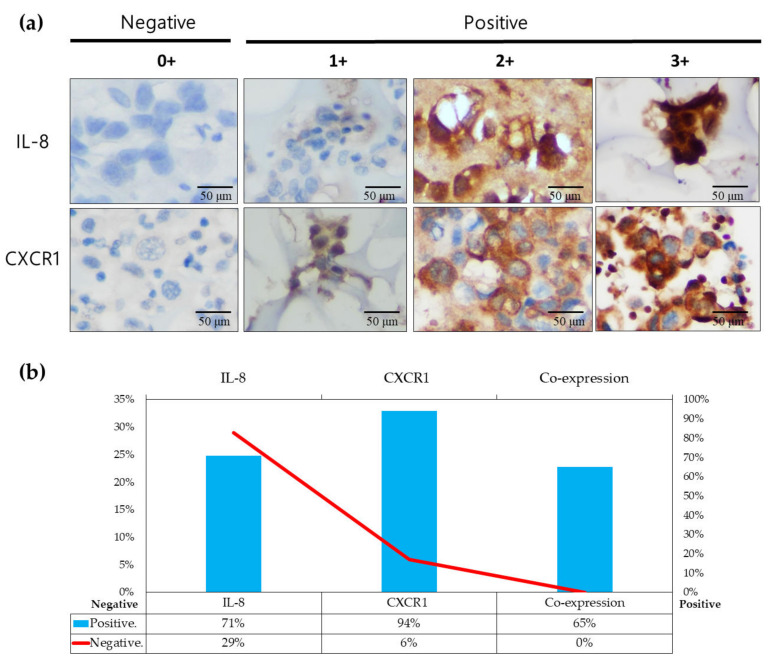
Validation of IL-8 and CXCR1 expression in MPE (**a**) Representative images of IL-8 and CXCR1 expression on cytology are shown, indicating absent (0), weak (1+), moderate (2+), and strong (3+) staining intensity (Scale bar: 50 μm). (**b**) The proportion of increased IL-8 expression in cell blocks of PADC with MPE was estimated to be 71%, whereas the corresponding CXCR1 expression in cell blocks of PADC with MPE was measured up to 94%. Moreover, the majority (11/17) of MPE cases showing co-expression of IL-8/CXCR1 accounted for 65% of the total.

Seventeen cases of PADC with MPE, including surgical specimens and cytology, underwent analysis for IL-8 and CXCR1. The intensities of IL-8 and CXCR1 were classified as 0, 1, 2+, or 3+ [22]. An intensity of 0 was regarded as negative, while intensities of 1+, 2+, and 3+ were considered positive (Figure 5a). Among the 17 cases, IL-8 and CXCR1 expression in MPE were observed in 71% (12/17) and 94% (16/17) of cases, respectively (Figure 5b). The co-expression of IL-8 and CXCR1 was present in 65% (11/17) of cases (Figure 5b). Co-expression of IL-8/CXCR1 in MPE was significantly higher than that in primary tumors, indicating the importance of IL-8/CXCR1 in FME. Among seventeen PADC cases, the individual proportion of PADC with MPE was measured at 53%, while those without MPE were estimated at 47%. However, 100% of patients with MPE eventually developed distant metastases, including lung to lung (66%), multiple seeding in pleura (55%), brain (44%), and bone (33%) (Table 1). In contrast, only 25% of patients without MPE subsequently developed distant metastasis (Table 1). Additionally, among the seventeen cases of PADC with MPE, 47% exhibited pleural invasion (PL1 and PL2), while 41% showed no signs of pleural invasion (Table 2). The individual percentage of PADC cases with pleural invasion (PL1 and PL2) eventually led to the development of 88% MPE and 75% metastases (Table 2). However, some cases of PADC without pleural invasion showed no signs of MPE, and only 28% developed metastases (Table 2). These data indicate that MPE serves as the primary route for early metastatic dissemination in PADC, emphasizing the significance of FME as a reservoir for distant metastasis, including those in the brain, bone, and other lung tissues (lung-to-lung metastasis).

## 4. Discussion

MPE, consisting of PADC cells with abundant parietal pleural lymphatic vessel drainage [25], indicates the initial pathological metastasis, which is a crucial step in the metastatic cascade [1]. Firstly, liquid effusions present as superior to solid-phase environments due to their enhanced capability to envelop tumor tissue, thereby guaranteeing a superior delivery of growth factors. Secondly, with the increasing shedding of soluble factors and ligands by tumor cells into their microenvironment, pleural effusion emerges as a more dynamic reservoir of these bioactive molecules. Additionally, it can readily expand in volume to meet the rising demands imposed by the continuous release of factors from the tumor cell population [26]. It is widely accepted that effusions may provide a premetastatic niche supplying cancer tissue with various growth factors, including Il-6, IL-8, and IL-β1 [27]. As the disease progresses, effusions can expand to accommodate the increasing tumor cell population’s demand for essential factors [26]. However, the molecular mechanisms underlying MPE tumor progression during the metastatic cascade remain poorly understood. Previous studies have indicated an increase in IL-8 expression in cancer patients with MPE, and higher serum IL-8 levels in PADC may be associated with exosomal release [28]. As mentioned earlier, our study was consistent with prior findings and revealed four times higher levels of IL-8 in MPE compared to those in serum, as illustrated in Figure 1a,b.

In our study, we initially observed abundant IL-8 in both serum and effusion of MPE compared to SDPE (Figure 1a,b). Primary cultured cells from metastatic PADC (P#2045) in MPE under specific FME conditions showed higher IL-8 expression than the lung cancer cell line (NCI-H1792) lacking a unique FME (Figure 1c,d). Subsequently, we treated two representative cell lines (P#2045 and NCI-H1792) with rIL-8 to simulate paracrine IL-8 signaling. This treatment confirmed IL-8’s role in enhancing EMT by promoting cell migration (Figure 2a,d), invasion (Figure 2b), and upregulating EMT markers (Figure 2c). Wound-healing assays demonstrate that rIL8 significantly enhances cell migration in both NCI-H1792 and P#2045 cell lines (Figure 2d,e), with effects intensifying over time. Particularly, the P#2045 cell line displayed a notable response to rIL8 at 24 h (Figure 2e), showcasing its heightened sensitivity to this stimulation. These results underscore IL8’s pivotal role in promoting cell migration and invasion, relevant to cancer progression and metastasis. Moreover, IL-8 augmented CSC properties, as evidenced by the increased tumorigenicity in vitro (Figure 3a), upregulated immunoexpression and protein expression of CSC markers (Figure 3b,c), enhanced radio-resistance (Figure 3d) and chemo-resistance (Figure 3e), and over-protein-expression of drug-resistant gene products (Figure 3f). Additionally, we identified the corresponding receptor for IL-8, CXCR1, and validated IL-8/CXCR1 autocrine signaling circuits in cell lines and primary cultured cells using immunocytochemistry (Figure 4a,b). Co-expression of IL-8/CXCR1 in most patients with MPE (65%, 11/17) was associated with distant metastases and poor outcomes (Figure 5). The presence of MPE in PADC was associated with a higher likelihood of distant metastasis compared to cases without MPE (100% vs. 25%, Table 1). Additionally, the majority of cases with pleural invasion and subsequent MPE development ultimately led to distant metastasis (Table 2). Collectively, special FME in MPE should be considered a reservoir for distant metastases

Once MPE develops, it signifies that tumor cells have proliferated within the pleural cavity, separating from their original anchorage on the basement membrane. This detachment marks the initial phase of metastasis. However, as these cells continue to invade new FME, they are confronted with additional adaptive barriers that must be overcome to further their metastatic journey [1,29,30]. The question stands as to why these detached tumor cells can uniquely proliferate and grow instead of anoikis, despite their presumably unfavorable milieu [31]. The answer is that effusions offer a premetastatic niche full of a variety of mediators, which can supply the cancer tissue through autocrine and/or paracrine interactions with growth factors [26]. Growth factors such as transforming growth factor beta and vascular endothelial growth factor, together with additional stimuli including chemokines and cytokines, are predominantly synthesized by cellular constituents, namely mesothelial cells, stromal cells, and inflammatory cells [32,33,34,35,36]. If the effusions accumulate, the FME appears critical for permitting the cancer cells to interact easily and sufficiently with their surroundings including different types of cells and lots of mediators, through perfusion rather than through the anatomical architecture of the blood flow [37]. When cancer cells infiltrate a novel microenvironment, they swiftly interact with the surrounding FME, inducing phenotypic alterations in these cells and transforming the environmental composition. This dynamic shift plays a pivotal role in driving disease advancement by promoting enhanced tumor proliferation, invasive behavior, and metastatic spread. As a result, the FME of the effusions may accelerate the tumor’s aggressiveness by triggering the properties of CSC and EMT. IL-8 affects proliferation, migration, invasion, and mobility of cancer cells, and may induce morphological and molecular changes associated with EMT by changes in shape from cuboidal to spindle, actin cytoskeleton remodeling, upregulation of vimentin, and downregulation of E-cadherin in lung carcinoma cells [6,7,38].

The TME is composed of various stromal cells, including inflammatory, immune, adipose, endothelial, and fibroblast cells. In contrast, the FME predominantly contains immune, inflammatory, and mesothelial cells. A critical subset within the cancer cell population, known as CSCs, is implicated in the processes of metastatic spread, self-renewal, and response to cancer therapies. The interrelationship between the FME and CSCs remains a complex and poorly understood aspect of metastasis and resistance to treatment in oncology [39]. The FME’s immune and inflammatory cells release various growth factors, cytokines, chemokines, microRNAs, and exosomes. These secretions are instrumental in facilitating the acquisition and preservation of CSC characteristics. This dynamic and complex interaction contributes to the evolution of cancer cells into more aggressive, invasive, and migratory phenotypes, thereby promoting cancer development [39]. This evolutionary process is critical in the formation of a distinct subset of CSCs known as migratory CSCs. These cells, characterized by enhanced migratory and invasive capabilities, are distinguished by their ability to initiate and drive the process of metastasis. They achieve this by detaching from the primary tumor, invading surrounding tissues, and traveling to distant sites where they can establish new tumor colonies. Increased expression of IL-8 in FME enhances CSC growth, migration, invasion, and EMT markers; promotes sphere-forming ability; and leads to overexpression of Nanog, OCT4, and CD133, contributing to therapeutic resistance [2]. The observed increase in IL-8 production within PADC cell lines cultured in 3D environments (just like our nonadhesive sphere culture system, see Section 2.9) following cisplatin treatment may induce a response promoting cytokine release. This hypothesis suggests a potential mechanism behind enhanced IL-8 expression in response to chemotherapy in three-dimensional cellular models [40]. Targeting IL8 as a sequential therapy strategy to overcome chemotherapy resistance in advanced gastric cancer demonstrates the efficacy of IL-8 inhibitors in overcoming resistance to chemotherapy in gastric cancer [41], which underscores the potential utility of similar strategies in MPE. Furthermore, IL-8 in the FME may subsequently trigger cancer cells to generate more IL-8, which reacts with CXCR1 on the tumor cell surface in a positive feedback loop, leading to cancer progression through such a vicious cycle.

**Figure 6:**
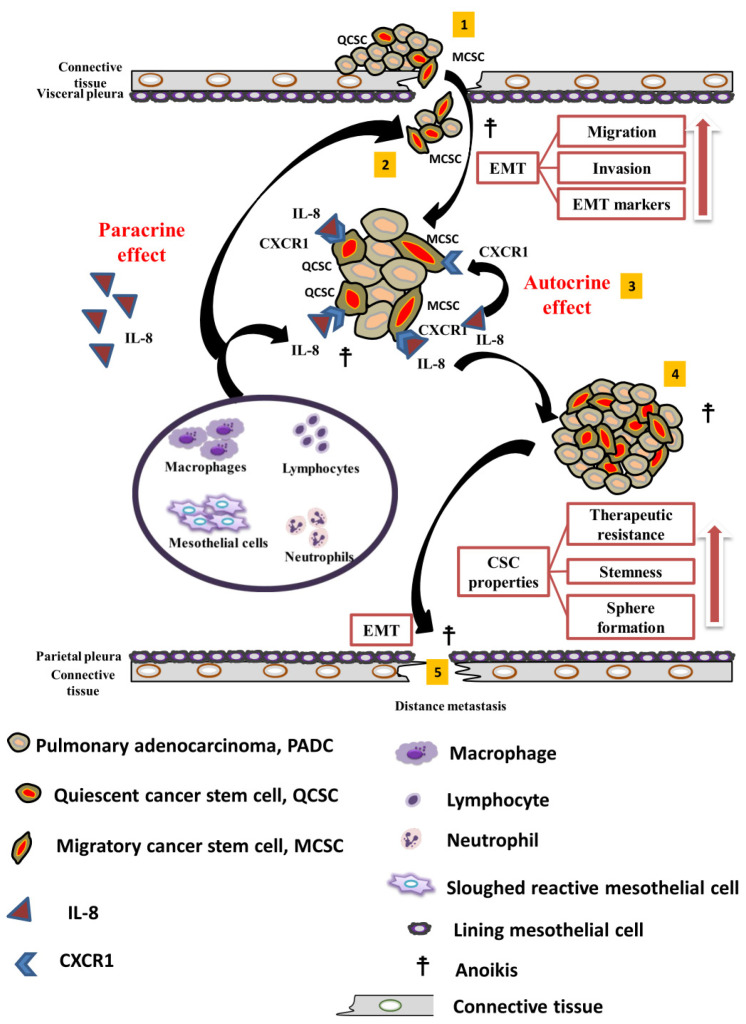
Diagrammatic illustration demonstrates a model depicting the paracrine and autocrine effects of IL8/CXCR1 signaling in MPE, which were divided into five sequential steps in the metastatic cascade. (1) PADCs are usually found in the peripheral areas at the edges of each lobe. PADC cells, consisting of a small portion of subset CSCs within the tumor, display traits akin to inactive or dormant cancer stem cells (CSCs), known as “quiescent CSCs, (QCSC)” when along with signs of EMT, a few of them become as active form also known as migratory CSCs (MCSCs), which show more aggressive behavior. These cells actively move and penetrate the connective tissue and visceral pleura, eventually leading to the pleural space and causing MPE. (2) Isolated or small clusters of PADC cells in MPE sometimes display micropapillary patterns or become smaller spheroids. To avoid the threat of anoikis, not only managing survival but also showing continuous proliferation which is due to the unique FME, particularly enriched with interleukin-8 (IL-8) produced by inflammatory cells such as lymphocytes, neutrophils, macrophages, and possibly mesothelial cells via paracrine effects. (3) Following the paracrine effect of IL-8 in conjunction with the CXCR1 receptor near a specialized premetastatic area on either MCSC or QCSC cell surfaces, self-stimulation, and proliferation by the IL-8/CXCR1 axis maintains cancerous tissue and ensures tumor cell survival in an autocrine manner. (4) Then to preserve and increase CSC properties, tumor nests become larger, and round clusters proliferate rapidly in IL-8 nutrient-rich effusion, leading to the development of more spheroids with increased cancer stemness and therapeutic resistance to chemotherapy, radiation, and drugs. (5) Finally, specific cells within these spheroids are stimulated by environmental signals, revive EMT and CSC characteristics, and evolve into metastatic CSCs. They then invade the nearby parietal pleura with rich angiolymphatic vessels, paving the way for further spreading to distant organs.

Based on our results, IL-8 emerges as a pivotal mediator in signaling and interacting with tumor cells, thus influencing the progression of advanced lung cancer and promoting the potential of CSCs in MPE. The major themes of our proposal are illustrated diagrammatically in Figure 6. The role of IL8/CXCR1 signaling in MPE can be delineated into the following five steps. (1) Migratory CSCs undergo a transition from quiescent CSCs in PADC to an active state as they infiltrate pleural tissues, contributing to the development of MPE. (2) During MPE, PADC must undergo anoikis; however, some surviving PADC cells proliferate owing to the presence of IL-8 from inflammatory cells and/or reactive mesothelial cells in the FME via a paracrine effect. (3) A premetastatic area rich in IL-8 supports tumor survival and self-proliferation to develop tumor stemness properties via paracrine/autocrine effects. (4) CSCs rich in IL-8/CXCR1 in the effusion form more spheroids and develop therapeutic resistance. (5) Stimulated CSCs evolve into metastatic CSCs that invade nearby tissues and vessels for further dissemination.

## 5. Conclusions

Our study confirmed that pleural effusion may provide a premetastatic niche for studying the cytokines involved in the microenvironment. The pivotal role of IL-8 expression in FME contributes to the enrichment of CSC properties via paracrine signaling IL-8 subsequently triggers cancer cells to generate IL-8 in an autocrine manner via CXCR1, resulting in cancer progression. These findings indicate that targeting the IL-8/CXCR1 signaling pathway could potentially reduce the aggressiveness of the disease and may provide a therapeutic approach to improve the prognosis of patients with MPE, although further research is needed to validate the hypothesis. Our future research will expand to include not only knockdown studies of IL-8 and its receptors CXCR1 and CXCR2 using siRNA but will also incorporate animal experiments and the recruitment of more patients. This comprehensive approach aims to validate the clinical significance of targeting IL-8/CXCR1 signaling as a potential therapeutic avenue. We intend to assess the efficacy of interventions targeting these pathways in both preclinical and clinical settings, thereby providing a robust evaluation of their potential as therapeutic options.

## Figures and Tables

**Table 1:** MPE in PADC was associated with a higher likelihood of distant metastasis.

PADC	Percentage	Metastasis	Metastatic Site	Percentage
With MPE	53% (9/17)	100% (9/9)	Lung	66% (6/9)
			Pleura	55% (5/9)
			Bone	44% (4/9)
			Brain	34% (3/9)
			Pericardium	11% (1/9)
			Peritoneum	11% (1/9)
Without MPE	47% (8/17)	25% (2/8)	Lung	100% (2/2)
			Pleura	50% (1/2)

**Table 2:** PADC with pleural invasion (PL) and subsequent MPE development ultimately led to distant metastasis.

Pleural Invasion	PL1/PL2	PL0
	47% (8/17) *	41% (7/17) *
MPE	88% (7/8)	0% (0/7)
Metastasis	75% (6/8)	28% (2/7)
Metastatic sites	Lung 50% (4/8) Pleura 50% (4/8) Brain 38% (3/8) Bone 25% (2/8)	Lung 28% (2/7) Pleura 14% (1/7)

* Two cases of pleural conditions were not available for assessment: PL1: pleural invasion 1, tumor invasion beyond the elastic layer; PL2: pleural invasion 2, tumor invasion to the parietal surface; PL0: tumor without pleural invasion.

## Data Availability

The data used in this study are available upon request from the corresponding author.

## Supplementary Materials

The following are available online at https://www.mdpi.com/article/10.3390/cells13110968/s1, Figure S1: Re-confirmation of the PADC cell line, P#2045, by two pathologists. Figure S2: Comparative analysis of IL-8 levels in pleural effusion and serum, a paired t-test on effusion samples (n = 25) and serum samples (n = 10). Figure S3: Testing of different dosages and time and determination of the most optimal rIL-8 concentration and reaction time. Table S1: Summary of clinical information in malignant MPE and SDPE. Table S2: Summary of clinical information in surgical specimens and cell blocks. Table S3: List of antibodies. Table S4: A paired t-test on effusion samples (n = 25) and serum samples (n = 10), Table S5: Wound-healing assay raw data.
