# Supplementary material for: Interleukin-8/CXCR1 Signaling Contributes to the Progression of Pulmonary Adenocarcinoma Resulting in Malignant Pleural Effusion
Source: Cells. 2024 Jun 3;13(11):968. doi: 10.3390/cells13110968 (PMC11172099; doi:10.3390/cells13110968)
Supplement: Supplementary file 1 [file cells-13-00968-s001.zip › cells-2945494-supplementary.pdf]

Table S1. Summary of clinical information in malignant pleural effusion (MPE) and systemic disease with pleural effusion (SDPE)

Case ID	Age	Gender	Diagnosis	TTF-1	TNM	AJCC 8th	Pleural effusion	Serum
Adenocarcinoma with MPE								
1	71	F	Adenocarconoma	Positive	T2N0M1a	4A	A	A
2	65	M	Adenocarconoma	Positive	T3N1M1c	4B	A	A
3	43	F	Adenocarconoma	Positive	T1N3M1c	4B	A	A
4	54	F	Adenocarconoma	Positive	T4N2M1a	4A	A	A
5	68	M	Adenocarconoma	Positive	T3N1M1a	4A	A	A
6	67	F	Adenocarconoma	Positive	TxNxM1a	4A	A	A
7	58	M	Adenocarconoma	Positive	T4N3M1c	4B	A	A
8	66	F	Adenocarconoma	Positive	T4N2M1c	4B	A	A
9	81	M	Adenocarconoma	Positive	T4N3M1a	4A	A	A
10	65	M	Adenocarconoma	Positive	T2bN0M1a	4A	A	A
11	54	M	Adenocarconoma	Positive	T4N2M1b	4A	A	A
12	53	M	Adenocarconoma	Positive	T4N2M1b	4A	A	A
13	89	F	Adenocarconoma	Positive	TxNxM1a	4A	A	A
14	86	F	Adenocarconoma	Positive	TxNxM1a	4A	A	A
15	68	M	Adenocarconoma	Positive	T3N1M1a	4A	A	A
16	53	M	Adenocarconoma	Positive	T4N3M1b	4A	A	A
17	73	F	Adenocarconoma	Positive	T4N3M1a	4A	A	NA
18	62	F	Adenocarconoma	Positive	T1aN2M1a	4A	A	NA
19	90	M	Adenocarconoma	Positive	T2aN2M1b	4A	A	NA
20	92	M	Adenocarconoma	Positive	T4NxM1a	4A	A	NA
21	70	M	Adenocarconoma	Positive	T4NxM1a	4A	A	NA
22	95	M	Adenocarconoma	Positive	T4N0M1a	4A	A	NA
23	57	F	Adenocarconoma	Positive	T3N2M1b	4A	A	NA
24	63	M	Adenocarconoma	Positive	T4N3M1a	4A	A	NA
25	47	F	Adenocarconoma	Positive	T1N0M1a	4A	A	NA
26	46	F	Adenocarconoma	Positive	T1aN0M1a	4A	A	NA
27	66	M	Adenocarconoma	Positive	T3N3M1b	4A	A	NA
28	61	M	Adenocarconoma	Positive	T4N3M1a	4A	A	NA
29	83	M	Adenocarconoma	Positive	T4N3M1a	4A	A	NA
30	70	M	Adenocarconoma	Positive	T4N3M1b	4A	A	NA
31	53	F	Adenocarconoma	Positive	T4N3M1a	4A	A	NA
32	48	F	Adenocarconoma	Positive	T2aN0M1a	4A	A	NA
33	84	M	Adenocarconoma	Positive	T2bN2M1b	4A	A	NA
SDPE								
1	91	F	Acute heart failure	NA	NA	NA	A	A
2	89	M	Valvular heart disease	NA	NA	NA	A	A
3	89	F	Chronic obstructive pulmonary disease	NA	NA	NA	A	A

4	82	M	Congestive heart failure	NA	NA	NA	A	A
5	94	M	Chronic kidney disease	NA	NA	NA	A	A
6	70	M	Acute kidney failure	NA	NA	NA	A	A
7	91	F	Valvular heart disease	NA	NA	NA	A	A
8	83	F	Congestive heart failure	NA	NA	NA	A	A
9	93	F	Valve heart disease	NA	NA	NA	A	A
10	53	M?	Cellulitis related sepsis	NA	NA	NA	A	A
11	76	M	Coronary artery disease	NA	NA	NA	A	A
12	74	M	Chronic heart failure	NA	NA	NA	A	A
13	92	F	Pulmonary embolism	NA	NA	NA	A	A
14	52	M	Liiver cirrhosis	NA	NA	NA	A	A
15	76	M	Type 2 diabetes mellitus with hyperosmolar hyperglycemic state	NA	NA	NA	A	A
16	80	F	Coronary artery disease	NA	NA	NA	A	A
17	72	F	Coronary atery disease	NA	NA	NA	A	A
18	80	F	Liver cirrhosis	NA	NA	NA	A	A
19	48	M	Liver cirrhosis	NA	NA	NA	A	A
20	58	M	Liver cirrhosis	NA	NA	NA	A	A
21	80	F	Coronary artery disease	NA	NA	NA	A	A
22	89	M	Acute respiratory distress syndrome	NA	NA	NA	A	A
23	54	M	Liver cirrhosis	NA	NA	NA	A	A
24	43	M	Liver cirrhosis	NA	NA	NA	A	NA
25	76	M	Liver cirrhosis	NA	NA	NA	A	NA

Abbreviations: A: available, NA: not available

Table S2. Summary of clinical information in surgical specimens and cell blocks

Case ID	Age	Gender	Diagnosis	TTF-1*	TNM	AJCC 8th when diagnosis	TD**	STAS	PL	Metastatic sites when diagnosis
1	M	66	Solid PADC	+	3A	T2bN2M0***	G3	1	0	Nil
2	F	34	Acinar PADC	+	3A	T1N2M0***	G3	1	0	Nil
3	F	53	Solid PADC	+	4A	T4N2M1a	G3	1	2	Pleura Lung
4	F	67	Papillary PADC	+	3A	T2aN3M0***	G3	1	0	Nil
5	M	67	Micropapillary PADC	+	4A	T3N0M1c	G3	**1**	**1**	Brain Pleura Pericardia Bone
6	F	54	Acinar PADC	+	4B	T3N0M1c	G3	1	1	Lung, Bone Brain
7	M	76	Micropapillary PADC	+	4A	T4N2M1b	G3	0	2	Lung, Bone
8	F	28	Solid PADC	+	4A	T2aN0M0***	G3	1	2	Nil
9	M	44	Micropapillary PADC	+	4A	T4N2M1a	G3	1	2	Pleura Lung Peritoneum
10	M	65	Solid PADC	+	3C	T3N3M0***	G3	1	0	Nil
11	F	57	Acinar PADC	+	3C	T3N3M0***	G2	0	2	Brain
12	M	77	Solid PADC	+	4A	T3N0M1a	G3	0	1	Pleura
13	F	64	Papillary PADC	+	4A	T4(m)N0M1a	G3	NA****	NA****	Lung
14	F	73	Cribriform PADC	+	4b	T2bN0M1c	G3	NA****	NA****	Lung Bone Pleura
15	F	60	Acinar PADC	+	1A	T1aN0M0***	G2	0	0	Lung
16	F	68	Micropapillary PADC	+	3A	T1bN2M0**	G3	1	0	Nil
17	F	48	Acinar PADC	+	3A	T1aN2M0***	G2	0	0	Lung Pleura

* indicates that immunohistochemical staining for TTF-1 includes both surgical specimens and cell blocks.
**Indicates the tumor differentiation, with G2 representing poorly differentiated components including solid, micropapillary, and complex glandular structures less than 20%, whereas G3 indicates over 20%.
*** indicates the patient had no MPE at initial diagnosis; and the MPE occurred more than **3** months later.
**** indicates this is a lung metastasis surgical specimen; the status of the primary tumor inlcuding STATS and PL are not available

Abbreviations: SS: Surgical Specimen, CB: Cell Block, TD: tumor differentiation, TNM: Tumor, Node, Metastasis, AJCC: American Joint Committee on Cancer, and STAS: Spread Through Air Spaces.

Table S3. List of antibodies

Antibody	Antibody subtype	Dilution	Manufacturer
TTF-1	Mouse monoclone	1:200	Dako
Nanog	Rabbit polyclone	1:50/1:1000	Abcam
OCT-4	Rabbit polyclone	1:250/1:000	Abcam
IL-8	Mouse monoclone	1:50/1:1000	Santa Cruz
CXCR1	Rabbit polyclone	1:100/1:1000	Novus
CD133	Rabbit monoclonal	1:200/1:1500	Abcam
E cadherin	Rabbit polyclone	1:3000	Gene Tex
N cadherin	Rabbit polyclone	1:2000	Gene Tex
Vimentin	Mouse monoclone	1:3000	Santa cruz
Snail	Rabbit monoclonal	1:1000	Abcam
Twist	Rabbit polyclone	1:1000	Cell Signalin
ABCG2	Rabbit monoclonal	1:1000	Abcam
MDR1	Rabbit monoclonal	1:1000	Abcam
Tubulin	Rabbit polyclone	1:5000	Cell Signalin

Table S4. A paired t-test on effusion samples (n=25) and serum samples (n=10). The statistical results show both p value less than 0.05 (p=0.001 and p=0.007).

Paired Sample T-Test

	Mean	N (Sample Size)	Standard Deviation	Standard Error of Mean
Paired 1 Pleural effusion (U/mL)<br>Pleural effusion (U/mL)	392.2243<br>80.450	25<br>25	379.03300<br>58.9035	75.80660<br>11.7807

Paired Sample Correlation

	N (Sample Size)	Correlation Coefficient	Significance (P-value)
Paired 1 Pleural effusion (U/mL) &<br>Pleural effusion (U/mL)	25	-.130	.534

Paired Samples T-Test

	Paired difference					T-Value	Degrees of Freedom	Significance (Two-tailed P-value)
				95% Confidence Interval				
	Mean	Standard Deviation	Standard Error of the Mean	Lower Bound	Upper Bound			
Paired 1 Pleural effusion (U/mL)-Pleural effusion (U/mL)	311.77465	391.10334	78.22067	150.33512	473.21417	3.986	24	.001

The IL-8 level of MPE and SDPE patients (n=25)

	Mean of paired differences	p-value
MPE_ Pleural effusion(U/mL)-<br>SDPE_ Pleural effusion(U/mL)	311.77	0.001

Paired Sample T-Test

	Mean	N (Sample Size)	Standard Deviation	Standard Error of Mean
Paired 1 Serum (U/mL)<br>Serum (U/mL)	79.733<br>2.140	10<br>10	70.2125<br>3.8018	22.2031<br>1.2022

Paired Sample Correlation

	N (Sample Size)	Correlation Coefficient	Significance (P-value)
Paired 1 Serum (U/mL) & Serum (U/mL)	10	.027	.942

Paired Samples T-Test

	Paired difference					T-Value	Degrees of Freedom	Significance (Two-tailed P-value)
				95% Confidence Interval				
	Mean	Standard Deviation	Standard Error of the Mean	Lower Bound	Upper Bound			
Paired 1 Serum (U/mL)- Serum (U/mL)	77.5922	70.2137	22.2035	27.3643	127.8200	3.495	9	.007

The IL-8 level of MPE and SDPE patients (n=10)

	Mean of paired differences	p-value
MPE_ Serum(U/mL)-<br>SDPE_ Serum(U/mL)	77.59	0.007

Table S5. Wound healing assay raw data.

							Migration				AVG		
NCI-H1792		0	12h	24h		0	12h	24h			0	12h	24h
1st	IL-8(-)	801	738	663		1	1.085366	1.208145		NCI-H1792 IL-8 (-)	1	1.107514	1.223583
	IL-8(+)	805	500	480		1	1.61	1.677083		NCI-H1792 IL-8 (+)	1	1.579409	1.667749
2nd	IL-8(-)	808	723	657		1	1.117566	1.229833					
	IL-8(+)	800	518	476		1	1.544402	1.680672					
3rd	IL-8(-)	805	719	653		1	1.119611	1.232772					
	IL-8(+)	803	507	488		1	1.583826	1.645492					
											AVG		
P#2045		0	12h	24h							0	12h	24h
1st	IL-8(-)	803	700	655		1	1.147143	1.225954		P#2045 IL-8 (-)	1	1.143627	1.23366
	IL-8(+)	818	523	411		1	1.564054	1.990268		P#2045 IL-8 (+)	1	1.556045	1.964117
2nd	IL-8(-)	801	707	647		1	1.132956	1.238022					
	IL-8(+)	808	527	421		1	1.533207	1.91924					
3rd	IL-8(-)	809	703	654		1	1.150782	1.237003					
	IL-8(+)	809	515	408		1	1.570874	1.982843					
											AVG		
P#2045 invasion front		0	12h	24h							0	12h	24h
1st	IL-8(-)	803	700	655		1	1.147143	1.225954		P#2045 IL-8 (-)	1	1.143627	1.23366
	IL-8(+)	818	257	3		1	3.182879	272.6667		P#2045 IL-8 (+)	1	3.20763	169.204
2nd	IL-8(-)	801	707	647		1	1.132956	1.238022					
	IL-8(+)	807	255	5		1	3.164706	161.4					
3rd	IL-8(-)	809	703	654		1	1.150782	1.237003					
	IL-8(+)	809	247	11		1	3.275304	73.54545					

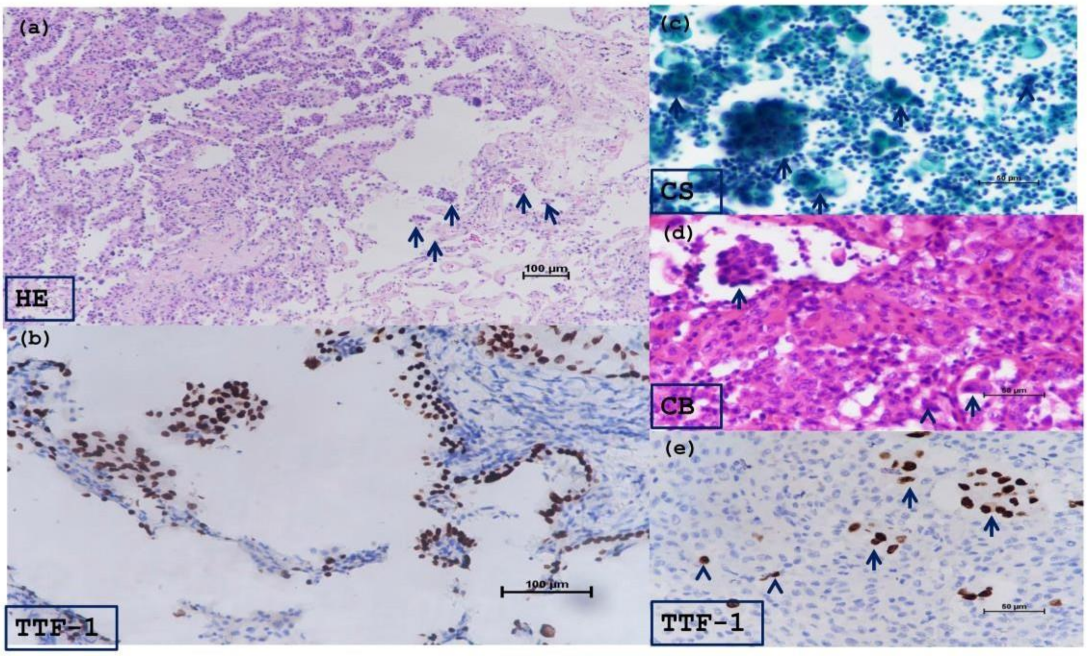

Figure S1. Re-confirmation of the PADC cell line, #P2045, by two pathologists.
(a) The tissue sections of representative cell line, #P2045, showed poorly differentiated PADC with micropapillary pattern and the spread through the air space, indicated by arrows (hematoxylin and eosin, magnification, ×100). (b) IHC confirmed the nuclear expression of TTF-1 indicative of micopapillary PADC (IHC, magnification, ×200). (c) The representative cell line was eventually chosen in our study owing to the grave outcome with transformation to MPE. The sections also showed similar micropapillary pattern with high N/C ratio, hyperchromatism, irregular membrane arranging in a 3-dimensional structure (smear after cytospin, Papanicolaou stain, magnification, ×400). (D) Further representative cell line in the cell block also showed similar morphology (cell block, hematoxylin and eosin, magnification, x200). (E) ICC reconfirmed the nuclear expression of TTF-1 in PADC with MPE (ICC, magnification, ×400)

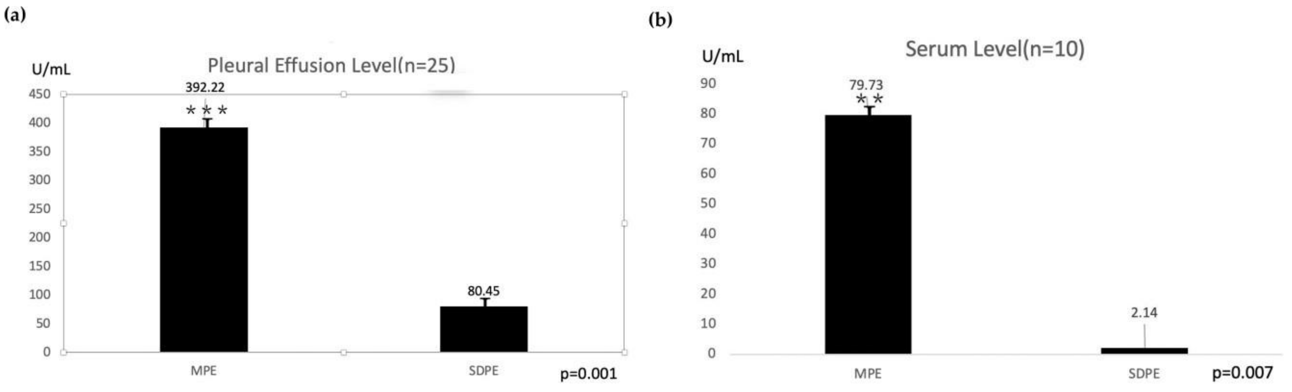

Figure S2. Comparative analysis of IL-8 levels in pleural effusion and serum, a paired t-test on effusion samples (n=25) and serum samples (n=10). (a) The IL-8 level in MPE is significantly elevated compared to SDPE, achieving statistical significance (p=0.001, paired t-test). (b) The IL-8 level in MPE is significantly higher than in SDPE, also reaching statistical significance (p=0.007, paired t-test) (Experiments were conducted in triplicate).

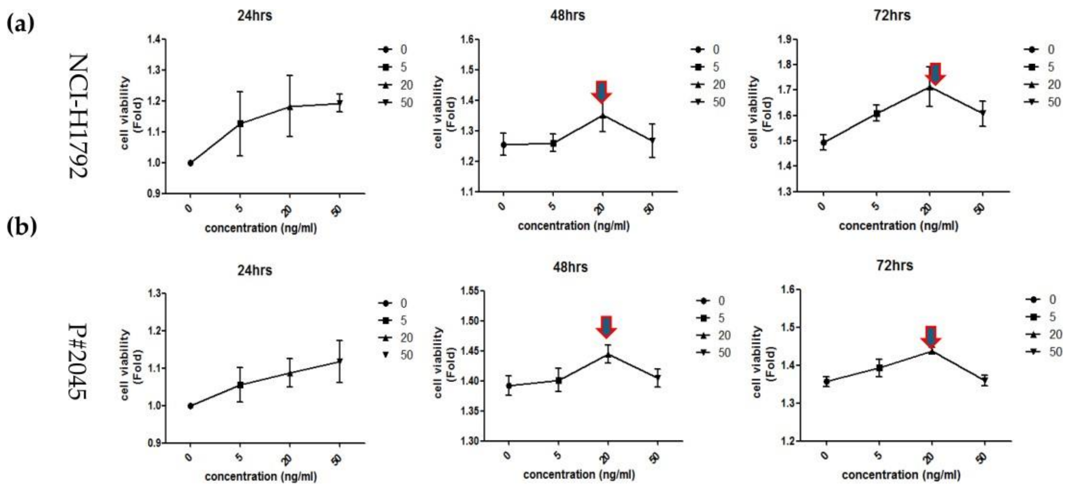

Figure S3. Testing of different dosages and time and determination of the most optimal rIL-8 concentration and reaction time. Experiments were conducted in triplicate. (a) NCI-H1792 was treated at various rIL-8 concentrations (0, 5, 20, and 50 μM), and cell viability was observed at different times (24, 48, 72 hours). The concentration of 20 μM and reaction time of 48 hours were chosen to validate whether IL-8 was able to increase the abilities of EMT and CSC properties. (b) The P#2045 was also performed in the same condition, which also showed similar results of the most optimal rIL-8 concentration and reaction time.
